# Selective Irreversible Inhibition of Neuronal and Inducible Nitric-oxide Synthase in the Combined Presence of Hydrogen Sulfide and Nitric Oxide*

**DOI:** 10.1074/jbc.M115.660316

**Published:** 2015-08-20

**Authors:** Christian L. Heine, Renate Schmidt, Kerstin Geckl, Astrid Schrammel, Bernd Gesslbauer, Kurt Schmidt, Bernd Mayer, Antonius C. F. Gorren

**Affiliations:** From the Departments of ‡ Pharmacology and Toxicology and; § Pharmaceutical Chemistry, Institute of Pharmaceutical Sciences, Karl Franzens University Graz, A-8010 Graz, Austria

**Keywords:** enzyme inactivation, hydrogen sulfide, nitric oxide, nitric-oxide synthase, reactive nitrogen species (RNS)

## Abstract

Citrulline formation by both human neuronal nitric-oxide synthase (nNOS) and mouse macrophage inducible NOS was inhibited by the hydrogen sulfide (H_2_S) donor Na_2_S with IC_50_ values of ∼2.4·10^−5^ and ∼7.9·10^−5^ m, respectively, whereas human endothelial NOS was hardly affected at all. Inhibition of nNOS was not affected by the concentrations of l-arginine (Arg), NADPH, FAD, FMN, tetrahydrobiopterin (BH4), and calmodulin, indicating that H_2_S does not interfere with substrate or cofactor binding. The IC_50_ decreased to ∼1.5·10^−5^ m at pH 6.0 and increased to ∼8.3·10^−5^ m at pH 8.0. Preincubation of concentrated nNOS with H_2_S under turnover conditions decreased activity after dilution by ∼70%, suggesting irreversible inhibition. However, when calmodulin was omitted during preincubation, activity was not affected, suggesting that irreversible inhibition requires both H_2_S and NO. Likewise, NADPH oxidation was inhibited with an IC_50_ of ∼1.9·10^−5^ m in the presence of Arg and BH4 but exhibited much higher IC_50_ values (∼1.0–6.1·10^−4^ m) when Arg and/or BH4 was omitted. Moreover, the relatively weak inhibition of nNOS by Na_2_S in the absence of Arg and/or BH4 was markedly potentiated by the NO donor 1-(hydroxy-*NNO*-azoxy)-l-proline, disodium salt (IC_50_ ∼ 1.3–2.0·10^−5^ m). These results suggest that nNOS and inducible NOS but not endothelial NOS are irreversibly inhibited by H_2_S/NO at modest concentrations of H_2_S in a reaction that may allow feedback inhibition of NO production under conditions of excessive NO/H_2_S formation.

## Teaser

**Background:** NO and H_2_S signaling pathways may be interdependent.

**Results:** Citrulline formation and NADPH oxidation by neuronal and inducible but not endothelial nitric-oxide synthase were inhibited irreversibly by H_2_S in the presence of NO.

**Conclusion:** NO synthase is isoform-specifically inactivated by a reaction product of NO and H_2_S.

**Significance:** Inhibition by NO/H_2_S may represent an autoregulatory feedback mechanism.

## Introduction

Nitric oxide (NO) and hydrogen sulfide (H_2_S) are two endogenously generated molecules that perform important functions in signal transduction (1–3). Nitric oxide is formed from l-arginine (Arg), molecular oxygen (O_2_), and NADPH-derived electrons in a reaction catalyzed by nitric-oxide synthase (NOS; EC 1.14.13.39). NOS is only active as a dimer and exists in three isoforms, neuronal, endothelial, and inducible NOS (nNOS,^2^ eNOS, and iNOS, respectively), that differ in tissue distribution and physiological function (4–6). The constitutive isozymes nNOS and eNOS are activated by Ca^2+^/calmodulin (CaM), whereas the much higher affinity of iNOS for CaM renders its activity [Ca^2+^]-independent under physiological conditions. Formation of NO requires the cofactor tetrahydrobiopterin (BH4), which couples NADPH oxidation to NO synthesis. In the absence of BH4, oxidation of NADPH results in O_2_^⨪^ formation (5, 7, 8).

In mammals, generation of H_2_S is catalyzed by cystathionine β-synthase (EC 4.2.1.22), cystathionine γ-lyase (EC 4.4.1.1), and 3-mercaptopyruvate sulfurtransferase (EC 2.8.1.2) (3, 9, 10). There is growing evidence that the NO and H_2_S signaling pathways are interdependent with both stimulatory and inhibitory effects being reported (2, 11–14). Although many of these effects appear to be indirect, there are some reports of direct effects of NO on the H_2_S-generating enzymes and of inhibition of NOS by H_2_S (15, 16). Furthermore, recent data suggest that reactions among NO, H_2_S, and their derivatives may be (patho)physiologically relevant. H_2_S as a reducing agent and nucleophile is predicted to react with a variety of NO-derived species, possibly yielding nitroxyl (HNO), thionitrous acid (HSNO), and nitrosopersulfide (SSNO^−^) as reaction products (9, 11–13, 17, 18).

In the present study, we investigated whether H_2_S is able to directly affect NOS activity. We found that recombinant human nNOS and murine iNOS but not human eNOS were irreversibly inhibited by modest (∼10^−5^ m) concentrations of H_2_S under conditions that allowed NO formation (*i.e.* +Arg/+BH4). In the absence of NO formation, inhibition required much higher H_2_S concentrations (∼10^−4^ m) and was reversed by dilution. The results suggest that a product of the reaction between NO and H_2_S, possibly SSNO^−^, irreversibly inhibits nNOS and iNOS. The potential physiological relevance of these observations is discussed.

## Experimental Procedures

### 

#### 

##### Materials

l-[2,3,4,5-^3^H]Arginine hydrochloride ([^3^H]Arg; 57 Ci/mmol) was from American Radiolabeled Chemicals Inc. purchased through Humos Diagnostic GmbH (Maria Enzersdorf, Austria). BH4 was from Dr. B. Schircks Laboratories (Jona, Switzerland). Stock solutions of BH4 were prepared in 10 mm HCl. Stock solutions of Na_2_S (Sigma-Aldrich, catalog number 407410) were prepared in Milli-Q water (Millipore; resistance, >18 megaohms·cm^−1^) and stored in dark vessels. General materials for molecular biology were from New England Biolabs; Life Technologies, Inc.; and Qiagen. The EasySelect^TM^ *Pichia* expression kit was from Invitrogen (Life Technologies, Inc.). Human nNOS cDNA was from Dr. John Parkinson (Berlex Biosciences, Richmond, CA). Purified yeast thioredoxin 1 and thioredoxin reductase were from Biomol (Sanova, Vienna, Austria). 1-(Hydroxy-*NNO*-azoxy)-l-proline, disodium salt (PROLI/NO) and spermine NONOate (SPER/NO) were from Enzo Life Sciences (Lausen, Switzerland). Disodium diazen-1-ium-1,2,2-triolate (Angeli's salt) was from Santa Cruz Biotechnology, Inc. (Heidelberg, Germany). NADPH was purchased from Pharma Waldhof GmbH (Düsseldorf, Germany). Glutathione persulfide was prepared as published (19) and used immediately. Other chemicals were from Sigma-Aldrich.

##### Enzyme Expression and Purification

Mouse macrophage iNOS was expressed in *Escherichia coli* and purified as described (20). Human eNOS was expressed in and purified from *Pichia pastoris* as described elsewhere (21). To subclone cDNA of human nNOS, the *P. pastoris* expression vector pPICZA was used (EasySelect *Pichia* expression kit). The plasmid pBBS230 containing cDNA for human nNOS was double digested with XbaI and NotI. The recessed 3′ termini from the XbaI digest were filled by the Klenow fragment of *E. coli* DNA polymerase I in the presence of appropriate deoxynucleoside triphosphates. The vector was subsequently double digested with EcoRI and after filling the recessed 3′ termini with NotI. The 4.3-kb insert was ligated to the restricted pPICZA. *E. coli* TOP10F′ cells were transformed with the resulting ligation products and plated on LB/Zeocin medium (1% tryptone, 0.5% yeast extract, 0.5% NaCl, and 25 μg/ml Zeocin at pH 7.5). The resulting transformants were tested by restriction analysis, and positive clones were amplified. The final DNA construct was linearized with PmeI, the DNA was transformed into *P. pastoris* GS115 (Mut^+^), and the cells were plated on YPDS/Zeocin medium (1% yeast extract, 2% peptone, 2% glucose, 1 m sorbitol, and 100 μg/ml Zeocin) to select recombinants. A single colony of the best clone was grown for 36 h at 30 °C in 50 ml of buffered minimal glycerol (BMGH) medium consisting of 100 mm potassium phosphate (pH 6.0), 13.4 g/liter yeast nitrogen base without amino acids, 400 μg/liter biotin, 40 mg/liter l-histidine, and 1% (v/v) glycerol. The overnight culture was diluted in BMGH medium (1:200) and grown overnight at 30 °C to an *A*_600_ of 5–6. To induce nNOS expression, cells were harvested and resuspended in the presence of 4 mg/liter hemin chloride in buffered minimal methanol medium consisting of 100 mm potassium phosphate (pH 6.0), 13.4 g/liter yeast nitrogen base without amino acids, 400 μg/liter biotin, 40 mg/liter l-histidine, and 0.5% methanol at an *A*_600_ of ∼1.

After 24 h of growth at 30 °C, cells were harvested by centrifugation at 2000 × *g* for 5 min at room temperature and resuspended at a concentration equivalent to an *A*_600_ of 125 (based on the *A*_600_ of the culture) in 50 mm Tris (pH 7.4) containing 1 mm EDTA, 5% glycerol, 12 mm 2-mercaptoethanol (2-ME), 1 mm phenylmethylsulfonyl fluoride (PMSF), and 1 mm CHAPS. An equal volume of glass beads (0.5 mm) was added to the suspension, and the cells were broken by vigorous vortexing at 4 °C for a total of 10 min in bursts of 30 s alternating with cooling on ice. The glass beads were separated by centrifugation at 800 × *g* for 5 min. After a further clearing step at 1600 × *g* for 5 min, the supernatant was centrifuged at 30,000 × *g* for 15 min. The enzyme was purified from the resulting supernatant by affinity chromatography as described previously (22). Final elution was achieved with 20 mm Tris (pH 7.4), 150 mm NaCl, and 4 mm EGTA. After determination of the protein concentration according to Bradford (23) using bovine serum albumin as a standard, the enzyme was stored at −70 °C in the presence of 1 mm CHAPS. Enzyme concentrations are expressed as the concentration of the monomer, assuming molecular masses of 160 (nNOS), 130 (iNOS), and 135 kDa (eNOS).

##### Determination of Enzyme Activity

NOS activity was determined as the formation of l-[^3^H]citrulline from [^3^H]Arg (24). Unless indicated otherwise, purified nNOS (5 μg/ml; 31.3 nm), iNOS (2 μg/ml; 15.4 nm), or eNOS (5 μg/ml; 37 nm) was incubated for 10 min in 0.1 ml of 50 mm triethanolamine HCl (TEA) (pH 7.4) containing 0.1 mm [^3^H]Arg (∼60,000 cpm), 0.2 mm NADPH, 5 μm FAD, 5 μm FMN, 10 μm BH4, 0.5 mm CaCl_2_, 10 μg/ml CaM, 0.2 mm CHAPS, 0.1 mm EDTA, and sodium sulfide (Na_2_S) as indicated at 37 °C followed by separation and detection of [^3^H]citrulline. Blank values were determined in the absence of enzyme. For activity measurements at varying pH values, 50 mm Bis-tris propane (pH 6.3–9.5) was used instead of TEA.

To test for irreversibility of inhibition, nNOS (250 μg/ml; 1.6 μm) was preincubated for 3 min in 0.1 ml of 50 mm TEA (pH 7.4) containing 0.1 mm Arg, 0.2 mm NADPH, 5 μm FAD, 5 μm FMN, 10 μm BH4, 0.5 mm CaCl_2_, 0.2 mm CHAPS, 0.1 mm EDTA, 300 μg/ml CaM, and 0.5 mm Na_2_S as indicated at 37 °C. After preincubation, samples were 50-fold diluted in prechilled buffer containing 50 mm TEA (pH 7.4), 0.2 mm CHAPS, and 0.1 mm EDTA in the absence or presence of thiol (2 mm DTT, 2 mm GSH, or 2.9 mm 2-ME). These mixtures were diluted 3-fold in 0.1 ml of 50 mm TEA (pH 7.4) containing 0.1 mm [^3^H]Arg (∼60,000 cpm), 0.2 mm NADPH, 5 μm FAD, 5 μm FMN, 10 μm BH4, 0.5 mm CaCl_2_, 10 μg/ml CaM, 0.2 mm CHAPS, and 0.1 mm EDTA in the absence or presence of 5 μm thioredoxin and 6 μm thioredoxin reductase followed by determination of [^3^H]citrulline formation at 37 °C for 10 min.

NADPH oxidation was determined spectrophotometrically at 340 nm and 37 °C as described elsewhere (25). Unless indicated otherwise, samples containing 10 μg/ml nNOS (62.5 nm), 0.2 mm NADPH, 0.5 mm CaCl_2_, 0.2 mm CHAPS, 0.1 mm EDTA, 0.1 mm Arg, 10 μm BH4, 30 μm PROLI/NO, and Na_2_S as indicated in 50 mm TEA (pH 7.4) were incubated at 37 °C. The reaction was initiated by the addition of 20 μg/ml CaM and monitored for 5 min. Rates were corrected by subtraction of blank rates obtained in the absence of CaM.

**FIGURE 1.**
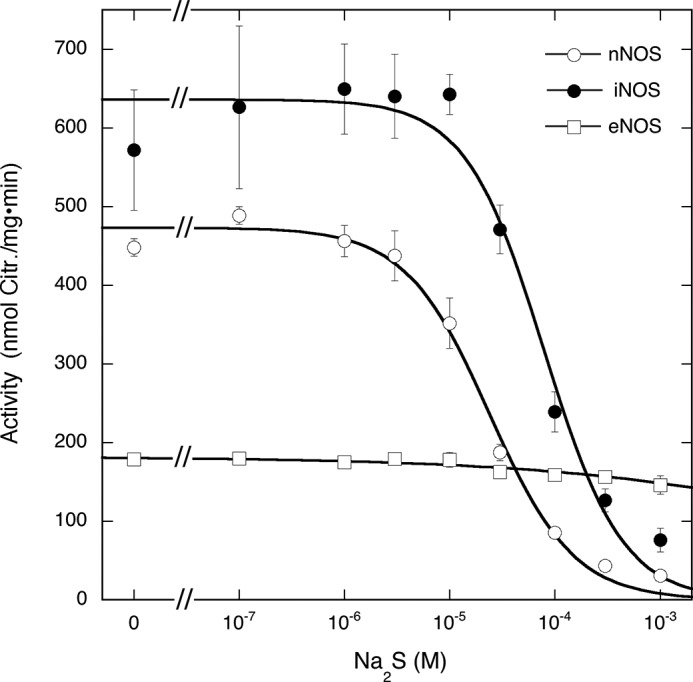
**Effect of Na_2_S on NOS-catalyzed citrulline formation.** The figure shows the effect of Na_2_S on citrulline (*Citr.*) formation by nNOS (*open circles*), iNOS (*closed circles*), and eNOS (*open squares*). Samples containing 0.2–0.5 μg of NOS, 0.1 mm [^3^H]Arg (∼60,000 cpm), 0.2 mm NADPH, 5 μm FAD, 5 μm FMN, 10 μm BH4, 0.5 mm CaCl_2_, 10 μg/ml CaM, 0.2 mm CHAPS, 0.1 mm EDTA, and Na_2_S as indicated in 0.1 ml of 50 mm TEA (pH 7.4) were incubated for 10 min at 37 °C. Data points (*n* = 3) are presented as mean values ±S.E. (*error bars*).

Concentration-effect curves (Figs. 1; 3; 4*A*; 7, *B* and *C*; 8, *A* and *B*; and 9*A*) were fitted to the Hill equation Act = Act_∞_ + (Act_0_ − Act_∞_)/(1 + ([*I*]/IC_50_)*^h^*) in which Act is the observed activity, [*I*] is the variable concentration of inhibitor (Na_2_S in Figs. 1, 3, 4, and 7; PROLI/NO in Fig. 8; and Angeli's salt in Fig. 9), Act_0_ and Act_∞_ are the respective activities at zero and infinite inhibitor concentration, IC_50_ is the half-maximal inhibitory concentration, and *h* is the Hill coefficient. Values for IC_50_, *h*, and Act_0_ and in Fig. 3 for Act_∞_ were determined from the fits; Act_∞_ was set to 0 in Figs. 1, 4, 7, 8, and 9. In Fig. 8*B*, *h* was set to 2.

The pH dependence of Fig. 4*B* was fitted to the equation IC_50_ = *K_i_*/(1 + 10^pH − p^*^Ka^*) where *K_i_* and p*K_a_* are the apparent inhibition constant of the protonated inhibitor and the corresponding acidity constant, respectively. This equation describes the dependence of the observed IC_50_ on pH when inhibition involves the protonated form only. The time traces in the presence of CaM of Fig. 7*A* were fitted to single exponential functions.

##### UV/Visible Absorbance Spectroscopy

Spectra were measured with a Hewlett-Packard 8452A diode array spectrophotometer. For absorbance measurements, nNOS or eNOS samples were diluted to a final concentration of approximately 4 μm in 50 mm TEA (pH 7.4) in the absence or presence of 5 mm NaHS.

##### Gel Filtration

NOS dimerization was analyzed by gel filtration with a Superose 6 HR 10/30 column under the control of an ÄKTA chromatography system at 8 °C. The flow rate was set to 0.3 ml·min^−1^, and the elution buffer consisted of 20 mm TEA (pH 7.4), 150 mm NaCl, 5% (v/v) glycerol, and 0.5 mm diethylene triamine pentaacetic acid. Purified nNOS (250 μg/ml; 1.6 μm) was incubated for 10 min in 0.4 ml of 50 mm TEA (pH 7.4) containing 0.1 mm Arg, 0.2 mm NADPH, 5 μm FAD, 5 μm FMN, 10 μm BH4, 0.5 mm CaCl_2_, 0.2 mm CHAPS, 0.1 mm EDTA, and 300 μg/ml CaM as indicated in the absence or presence of 0.1 mm Na_2_S at 37 °C. Subsequently, 80 μl of ice-cold EGTA (30 mm) was added, and samples were immediately frozen in liquid nitrogen. After thawing, 250-μl aliquots (50 μg of protein) were injected and monitored by UV/visible absorption at 280 nm.

##### Low Temperature Polyacrylamide Gel Electrophoresis-Western Blotting Analysis

Dimerization was also analyzed by low temperature PAGE (26) followed by Western blotting. Purified nNOS (5 μg/ml; 31.3 nm) or eNOS (5 μg/ml; 37 nm) was incubated for 10 min in 0.1 ml of 50 mm TEA (pH 7.4) containing 0.1 mm Arg, 0.2 mm NADPH, 5 μm FAD, 5 μm FMN, 10 μm BH4, 0.5 mm CaCl_2_, 0.2 mm CHAPS, and 0.1 mm EDTA in the absence or presence of 10 μg/ml CaM and 0.3 mm Na_2_S at 37 °C. Reactions were terminated by the addition of 0.1 ml of chilled 0.125 m Tris (pH 6.8) containing 4% (w/v) SDS, 10% (v/v) 2-ME, 20% (w/v) glycerol, and 0.02% (w/v) bromphenol blue. Samples containing 50 ng of nNOS or eNOS were subjected to SDS-PAGE for 100 min at 100 V on discontinuous 4% SDS gels (1.5 mm) using the Mini-Protean II system from Bio-Rad. Gels and buffers were equilibrated at 4 °C, and the buffer tank was cooled during electrophoresis in an ice bath. Separated proteins were transferred to nitrocellulose membranes (0.45 μm) by electroblotting at 240 mA for 110 min followed by immunodetection with anti-nNOS or anti-eNOS antibodies (1:1000 or 1:2000 dilution, respectively; BD Transduction Laboratories) using horseradish peroxidase-conjugated anti-mouse IgG (1:5000; BD Transduction Laboratories) and ECL detection reagent (Biozym, Hessisch Oldendorf, Germany). Immunoreactive bands were quantified densitometrically using E.A.S.Y. 1.3 Win 32 (Herolab, Vienna, Austria) and ImageJ 1.46r software (Wayne Rasband, National Institutes of Health).

## Results

### 

#### 

##### Effect of Na_2_S on Citrulline Formation by nNOS, iNOS, and eNOS

To determine the effect of H_2_S on NOS activity, we measured citrulline formation by the NOS isoforms in the presence of varying concentrations of Na_2_S. As illustrated in Fig. 1, Na_2_S inhibited nNOS and iNOS with IC_50_ values of (2.4 ± 0.3)·10^−5^ and (7.9 ± 1.6)·10^−5^ m, respectively, whereas eNOS was only marginally affected.

##### Effect of Na_2_S on the Optical Absorbance Spectra of nNOS

**FIGURE 2.**
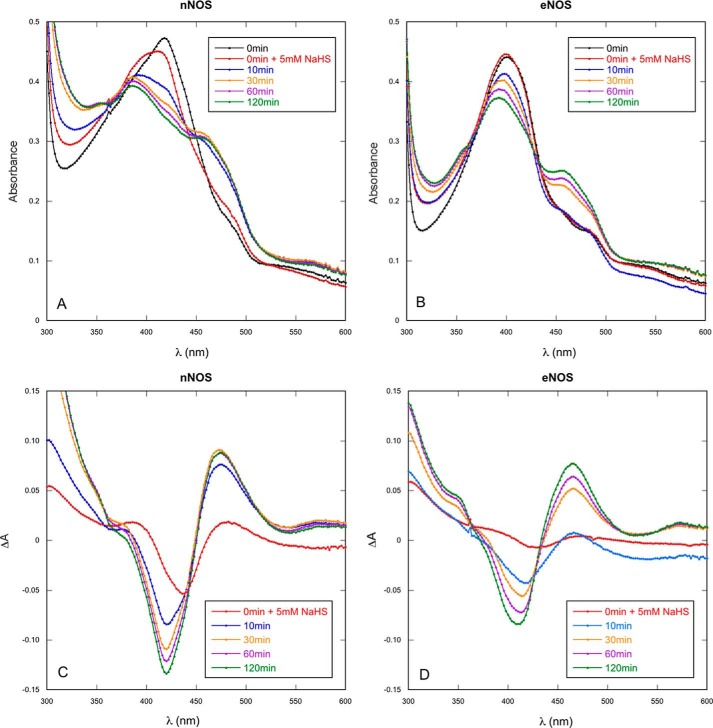
**Effect of Na_2_S on the UV/visible absorbance spectra of nNOS and eNOS.** The enzyme (nNOS or eNOS) was diluted to a final concentration of ∼4 μm in 50 mm TEA (pH 7.4). At time 0, NaHS (5 mm) was added, and spectra were measured at the indicated times. *A* and *B* show the absolute absorbance spectra of nNOS and eNOS, respectively. *C* and *D* show the corresponding difference spectra with the spectrum before Na_2_S addition subtracted from all other spectra.

Because it has been demonstrated that DTT and other thiols inhibit NOS by binding to the heme (27), we measured the effect of Na_2_S on the UV/visible absorbance of nNOS and eNOS. We observed spectral changes typical of the conversion to a thiol complex (Fig. 2). However, the transition was slow (*t*_½_ = 3.5 ± 1.6 min for nNOS), incomplete (approximately 50%), and required high concentrations (5 mm) of the H_2_S donor, suggesting that binding of the thiol to the heme is not involved in NOS inhibition.

##### Effect of Substrate and Cofactor Concentration on Inhibition of Citrulline Formation by Na_2_S

**FIGURE 3.**
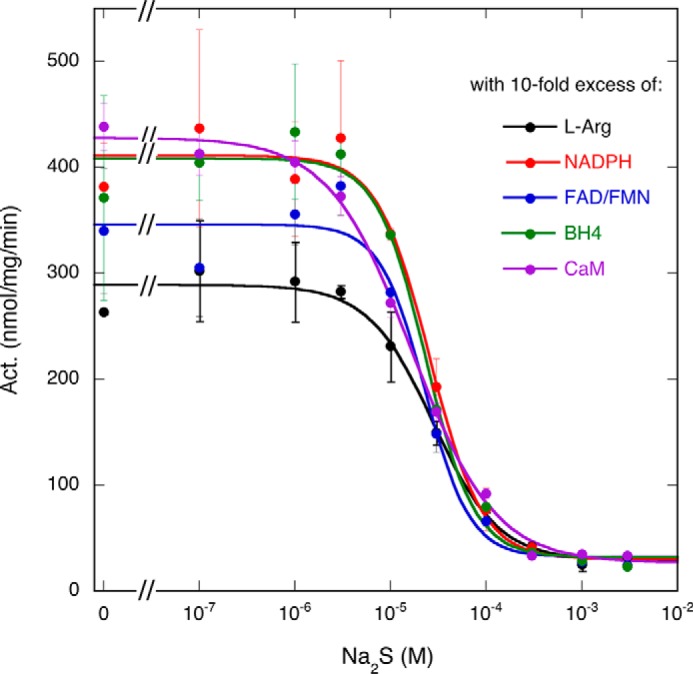
**Effect of excess Arg, NADPH, FAD/FMN, BH4, or CaM on Na_2_S-induced inhibition of nNOS-catalyzed citrulline formation.** The figure shows the effect of 10-fold excess Arg, NADPH, FAD/FMN, BH4, or CaM on inhibition by Na_2_S. Activity (*Act.*) was measured under the same experimental conditions as in Fig. 1 except that 1 mm Arg (*black circles*), 2 mm NADPH (*red circles*), 50 μm FAD/FMN (*blue circles*), 100 μm BH4 (*green circles*), or 100 μg/ml CaM (*violet circles*) was present. Data points (*n* = 2) are presented as mean values ±S.E. (*error bars*).

To investigate whether H_2_S inhibition is competitive with substrates or cofactors, citrulline formation by nNOS was examined in the presence of 10-fold higher concentrations of Arg, NADPH, FAD/FMN, BH4, or CaM. IC_50_ values were not affected by higher concentrations of these compounds (IC_50_ values: with 1 mm Arg, (2.8 ± 0.4)·10^−5^ m; with 2 mm NADPH, (2.6 ± 0.4)·10^−5^ m; with 50 μm FAD and 50 μm FMN, (2.3 ± 0.4)·10^−5^ m; with 100 μm BH4, (2.3 ± 0.3)·10^−5^ m; with 100 μg/ml CaM, (1.7 ± 0.2)·10^−5^ m; Fig. 3), which indicates that H_2_S does not interfere with substrate or cofactor binding.

##### Effect of Thiols on Na_2_S-induced Inhibition of Citrulline Formation by nNOS

Because H_2_S can modulate enzyme function by sulfhydration of cysteine residues (9–11, 13, 17, 28), it is conceivable that inhibition might be relieved in the presence of excess thiols. Therefore, we measured inhibition by Na_2_S in the presence of 2 mm DTT, 2 mm GSH, or 2.9 mm 2-ME. However, none of these thiols had any impact on IC_50_ values (results not shown).

##### Effect of Glutathione Persulfide on Citrulline Formation by nNOS

A potential complication is the facile formation of persulfides (RSS^−^) from H_2_S in the presence of thiols (2, 9–11, 17, 18). To study the possible involvement of persulfides in H_2_S-mediated inhibition of nNOS, we determined the effect of glutathione persulfide (GSSH) on nNOS activity. GSSH, synthesized from Na_2_S and GSSG according to a published procedure (19), inhibited nNOS with lower affinity than Na_2_S (IC_50_ = (1.16 ± 0.11)·10^−4^ m, *n* = 2; not shown). Because the conversion of GSSG and Na_2_S to GSSH by the applied method amounts to about 30–40% (19), the observed inhibition was most likely due to the remaining H_2_S. This suggests that persulfides do not significantly contribute to nNOS inhibition.

##### Effect of pH on Na_2_S-induced Inhibition of Citrulline Formation by nNOS

**FIGURE 4.**
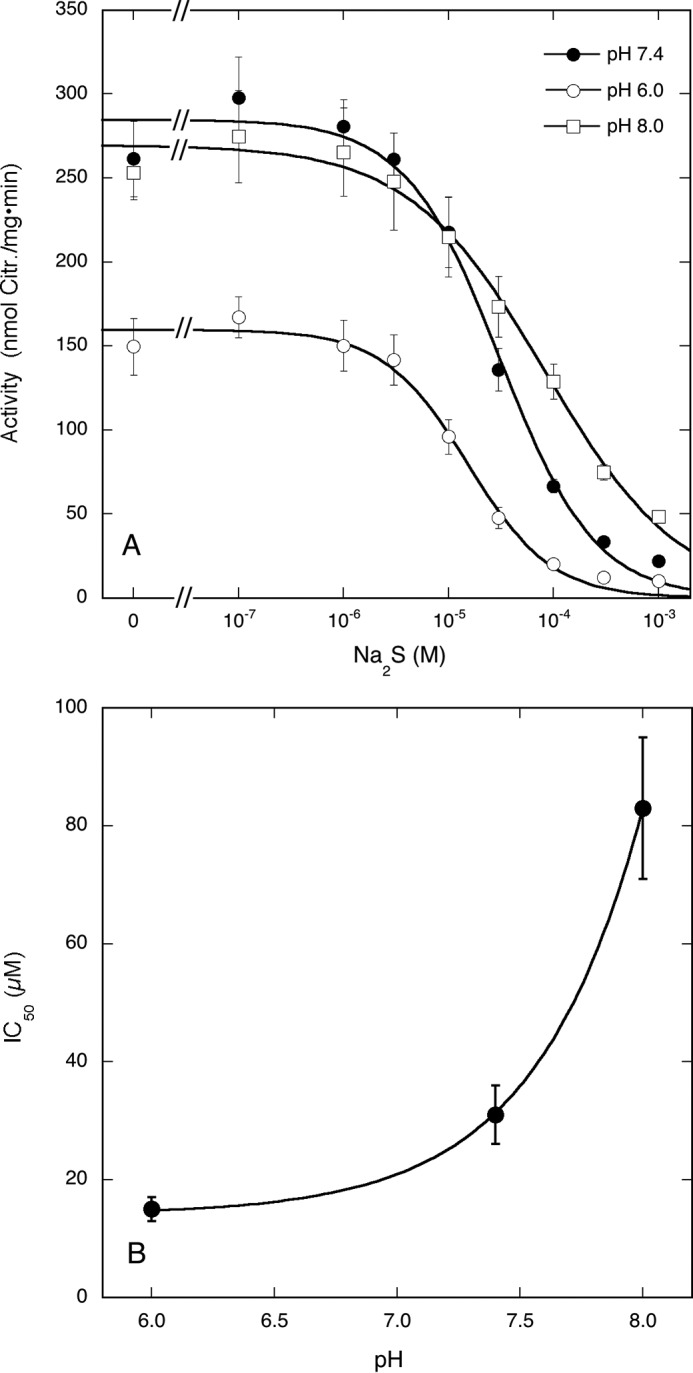
**Effect of pH on Na_2_S-induced inhibition of citrulline formation by nNOS.** *A* shows the inhibition of nNOS-catalyzed citrulline (*Citr.*) formation by Na_2_S at pH 7.4 (*closed circles*), 6.0 (*open circles*), and 8.0 (*open squares*). Samples containing 0.5 μg of NOS, 0.1 mm [^3^H]Arg (∼60,000 cpm), 0.2 mm NADPH, 5 μm FAD, 5 μm FMN, 10 μm BH4, 0.5 mm CaCl_2_, 10 μg/ml CaM, 0.2 mm CHAPS, 0.1 mm EDTA, and Na_2_S as indicated in 0.1 ml of 50 mm Bis-tris propane (pH as indicated) were incubated for 10 min at 37 °C. Data points (*n* = 3–4) are presented as mean values ±S.E. (*error bars*). *B* shows the IC_50_ values as a function of pH.

To study the effect of pH on Na_2_S-induced inhibition of citrulline formation, we determined the activity of nNOS at pH 6.0, 7.4, and 8.0 (Fig. 4*A*). The IC_50_ increased when the pH was raised from (1.5 ± 0.2)·10^−5^ m at pH 6.0 via (3.1 ± 0.5)·10^−5^ m at pH 7.4 to (8.3 ± 1.2)·10^−5^ m at pH 8.0. At first sight, these results suggest that Na_2_S-induced inhibition involves interaction of nNOS with H_2_S rather than with hydrogen sulfide anion (HS^−^). However, from a plot of IC_50_ against pH assuming inhibition by the low pH species only, we obtained a p*K_a_* value of 7.310 ± 0.014 (Fig. 4*B*), which is considerably higher than the published p*K_a_* (6.76 at 37 °C) of the H_2_S/HS^−^ equilibrium (29). The pH profile of inhibition therefore appears to reflect the protonation state of another compound.

##### Irreversible Inhibition by Na_2_S under Turnover Conditions

**FIGURE 5.**
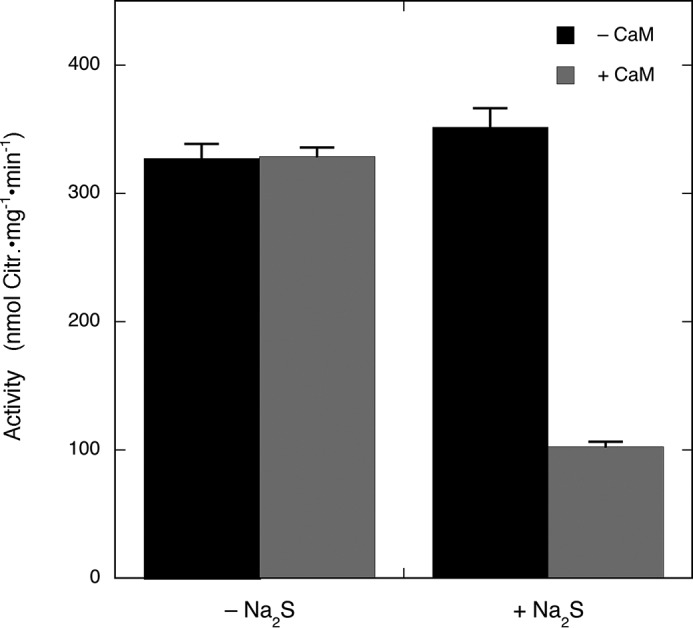
**Reversibility of nNOS inhibition after Na_2_S preincubation.** Neuronal NOS was preincubated with or without Na_2_S in the absence or presence of CaM. The figure shows citrulline (*Citr.*) formation by nNOS after 150-fold dilution of samples that were preincubated in the absence or presence of CaM and Na_2_S. See “Experimental Procedures” for details. Samples containing nNOS (250 μg/ml; 1.6 μm), 0.1 mm Arg, 0.2 mm NADPH, 5 μm FAD, 5 μm FMN, 10 μm BH4, 0.5 mm CaCl_2_, 0.2 mm CHAPS, 0.1 mm EDTA, 300 μg/ml CaM, and 0.5 mm Na_2_S as indicated in 0.1 ml of 50 mm TEA (pH 7.4) were preincubated for 3 min at 37 °C. After dilution, samples containing nNOS (1.7 μg/ml; 10.6 nm), 0.1 mm [^3^H]Arg (∼60,000 cpm), 0.2 mm NADPH, 5 μm FAD, 5 μm FMN, 10 μm BH4, 0.5 mm CaCl_2_, 10 μg/ml CaM, 0.2 mm CHAPS, and 0.1 mm EDTA in 0.1 ml of 50 mm TEA (pH 7.4) were incubated for 10 min at 37 °C. Assays also contained ∼3 μm Na_2_S carried over from the preincubation mixture. Data (*n* = 3–4) are presented as mean values ±S.E. (*error bars*).

**FIGURE 6.**
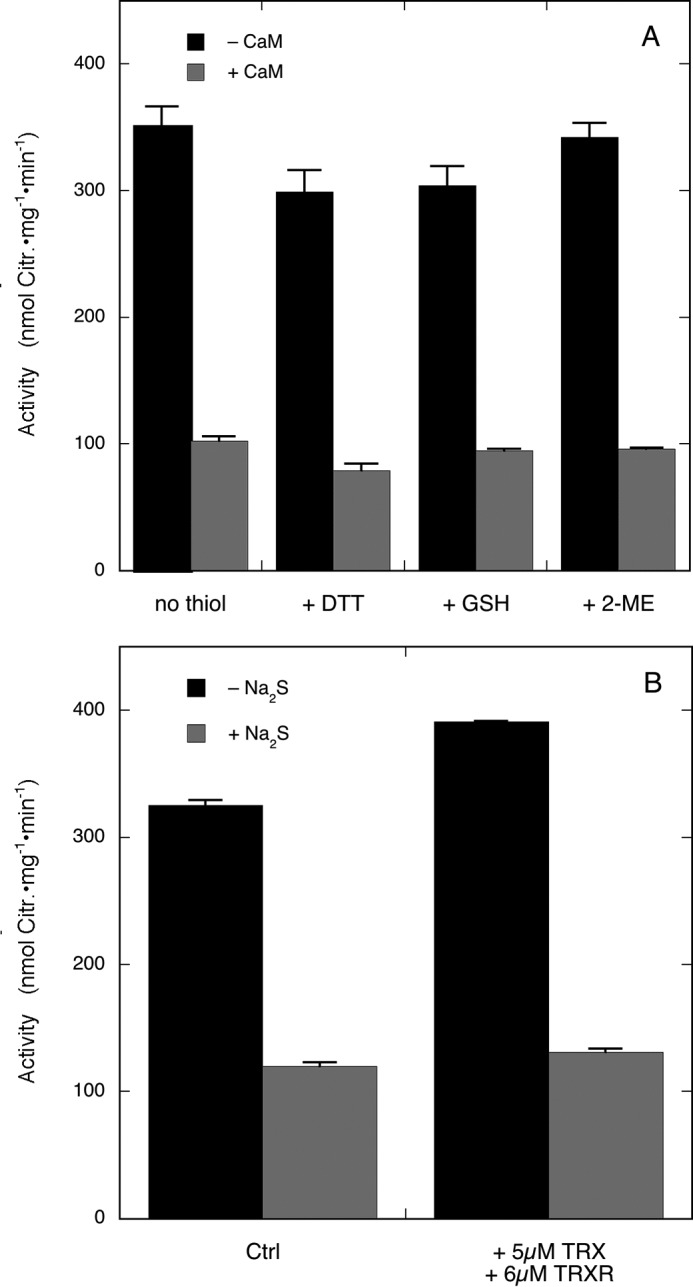
**Effect of thiols and thioredoxin/thioredoxin reductase on inhibition of nNOS by Na_2_S.** *A* shows the effect of various thiols on the reversibility of inhibition of nNOS by Na_2_S. Citrulline (*Citr.*) formation by nNOS was determined after preincubation in the absence or presence of Na_2_S and CaM. Experimental conditions were the same as for Fig. 5 except that thiol (2 mm DTT, 2 mm GSH, or 2.9 mm 2-ME) was added to the 50-fold diluted reaction mixture (see “Experimental Procedures”). Note that the final mixtures contained ∼3 μm Na_2_S carried over from the preincubation mixture and ∼0.7 mm DTT, ∼0.7 mm GSH, or ∼1 mm 2-ME carried over from the 50-fold diluted samples. Data (*n* = 2) are presented as mean values ±S.E. (*error bars*). *B* shows the effect of inclusion of thioredoxin/thioredoxin reductase on the reversibility of inhibition of nNOS by Na_2_S. Citrulline formation by nNOS was determined after preincubation in the absence or presence of Na_2_S. Experimental conditions were as for Fig. 5 except for the presence of thioredoxin reductase (*TRXR*) (6 μm) and thioredoxin-1 (*TRX*) (5 μm) during the final assay. Note that the final assay samples also contained ∼3 μm Na_2_S carried over from preincubation mixture. Data (*n* = 2) are presented as mean values ±S.E. (*error bars*). *Ctrl*, control.

As illustrated in Fig. 5, preincubation of nNOS with Na_2_S under turnover conditions decreased the activity after dilution (with ∼3.3 μm Na_2_S remaining) by approximately 70%, suggesting that inhibition by H_2_S is irreversible. However, the activity of the diluted enzyme was not affected when CaM was omitted during preincubation, which suggests that irreversible inhibition requires the presence of both H_2_S and NO. To elucidate whether thiols could reverse inhibition, we added 2 mm DTT, 2 mm GSH, or 2.9 mm 2-ME to the activity assay. As shown in Fig. 6*A*, none of these thiols restored the activity. Similarly, neither bovine serum albumin (2 mg/ml; data not shown) nor thioredoxin/thioredoxin reductase (Fig. 6*B*) reversed inhibition.

Similar observations were made with iNOS (not shown): preincubation in the absence and presence of 0.5 mm Na_2_S yielded activities after dilution of 528 ± 75 and 257 ± 45 nmol of citrulline/mg/min when CaM was present during preincubation, whereas the corresponding activities were 569 ± 65 and 617 ± 90 nmol/mg/min when CaM was omitted. As with nNOS, virtually identical results were obtained when 2 mm GSH was added to the assay mixture.

##### Effect of the Enzyme Concentration on Inhibition of nNOS and eNOS by H_2_S under Turnover Conditions

Because eNOS has lower turnover than nNOS and iNOS, the lack of inhibition of eNOS might be caused by the lower NO formation rate of that isoform. To explore that possibility, we determined the effect of the concentration of nNOS and eNOS (between 0.5 and 15.0 μg/ml and between 2.0 and 30.0 μg/ml, respectively) on the inhibition by 0.5 mm Na_2_S (Table 1). Both isoforms exhibited constant specific activities over the studied concentration range. However, whereas nNOS activity was almost completely (∼90%) blocked at all concentrations, eNOS activity was hardly affected even though the estimated NO formation rate (in the absence of Na_2_S) for the highest concentration of eNOS was 20× as high as that for the lowest concentration of nNOS. In the presence of Na_2_S, 30 μg/ml eNOS produced ∼160× as much NO as 0.5 μg/ml nNOS did. These results clearly demonstrate that the lack of inhibition of eNOS is not due to its lower intrinsic activity.

**TABLE 1:** **Effect of the enzyme concentration on H_2_S-induced inhibition of nNOS and eNOS** Conc is the enzyme concentration; Act_0_ and Act_H2S_ are the specific activities measured as citrulline formation in the absence and presence of 0.5 mm H_2_S, respectively; *v*_NO_^0^ and *v*_NO_^H2S^ are the corresponding NO formation rates in μm/min. See the legend to Fig. 1 for other experimental conditions.

Isoform	Conc	Act_0_	*v*_NO_^0^	Act_H2S_	*v*_NO_^H2S^	Inhibition
	μ*g*/*ml*	*nmol*/*mg*/*min*	μ*m*/*min*	*nmol*/*mg*/*min*	μ*m*/*min*	%
nNOS	0.5	383 ± 36	0.19 ± 0.02	53 ± 26	0.026 ± 0.013	86 ± 7
	2.0	405 ± 63	0.81 ± 0.13	31 ± 8	0.062 ± 0.016	92 ± 2
	10.0	306 ± 10	3.06 ± 0.10	24 ± 2	0.24 ± 0.02	92 ± 1
	15.0	309 ± 25	4.6 ± 0.4	29 ± 2	0.44 ± 0.03	91 ± 1
eNOS	2.0	129 ± 22	0.26 ± 0.04	98 ± 12	0.20 ± 0.02	24 ± 16
	10.0	160 ± 14	1.60 ± 0.14	134 ± 16	1.34 ± 0.16	16 ± 12
	15.0	153 ± 11	2.30 ± 0.17	146 ± 19	2.2 ± 0.3	5 ± 14
	30.0	128 ± 23	3.8 ± 0.7	136 ± 38	4.1 ± 1.1	−6 ± 35

##### Effect of Na_2_S on NADPH Oxidation by nNOS

**FIGURE 7.**
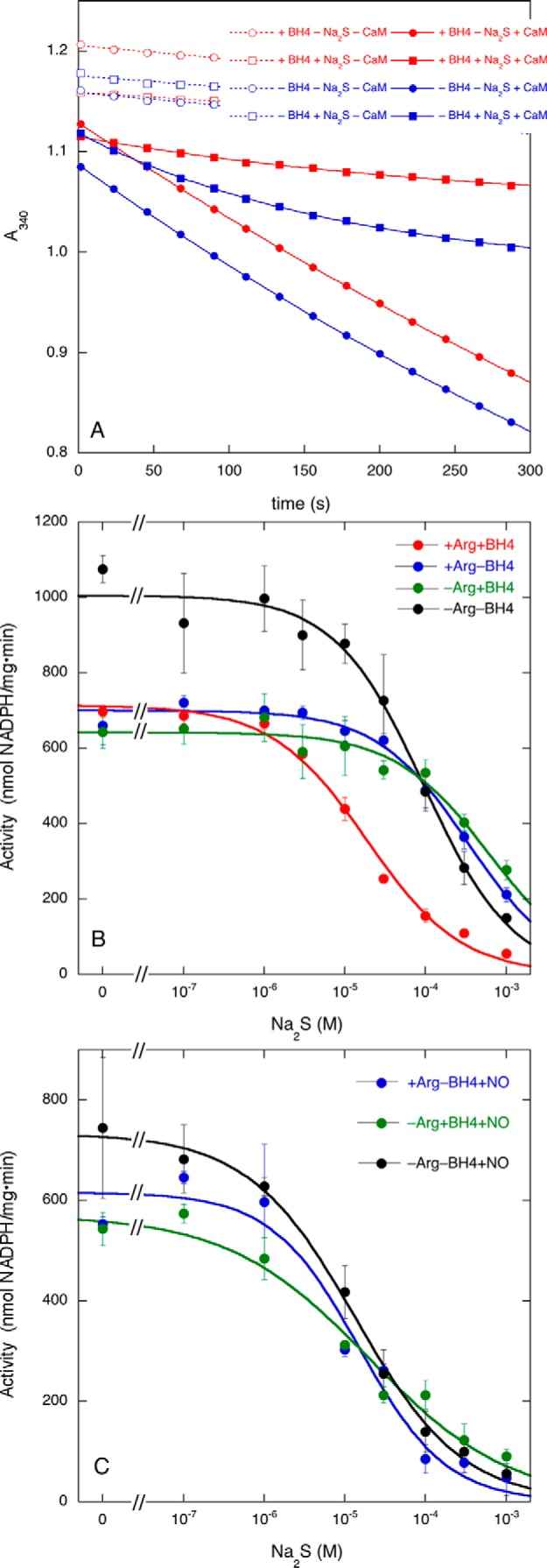
**Effect of Na_2_S on NADPH oxidation by nNOS in the absence or presence of NO.** *A* shows representative NADPH oxidation traces as measured by the absorbance decrease at 340 nm. Activity was determined in the presence (*closed symbols*, *continuous lines*) or absence (*open symbols*, *dotted lines*; “blanks”) of 20 μg/ml CaM in the presence (*red symbols* and *lines*; NO-producing conditions) or absence (*blue symbols* and *lines*; non-NO-producing conditions) of 10 μm BH4 and in the absence (*circles*; uninhibited traces) or presence (*squares*; inhibited traces) of 1 mm Na_2_S. In the experiments shown in *A*, Arg was always present, but similar experiments were performed in the absence of Arg (not shown). *B* shows the effect of Na_2_S on nNOS-catalyzed NADPH oxidation in the absence or presence of Arg and/or BH4. *C* shows the effect of inclusion of the NO donor PROLI/NO in the assay mixture. Samples (0.25 ml) contained 2.5 μg of NOS, 0.2 mm NADPH, 0.5 mm CaCl_2_, 20 μg/ml CaM (except for *A*, *open symbols*, *dotted lines*), 0.2 mm CHAPS, and 0.1 mm EDTA in 50 mm TEA (pH 7.4) and were incubated for 5 min at 37 °C; 0.1 mm Arg, 10 μm BH4, 30 μm PROLI/NO, and Na_2_S at varying concentrations (1 mm in *A*) were present as indicated. The reaction was initiated by the addition of CaM. Data (*n* ≥ 2) are presented as mean values ±S.E. (*error bars*).

To examine whether inhibition of citrulline formation was accompanied by NOS uncoupling, we determined the effect of Na_2_S on the rate of NADPH oxidation in the absence or presence of Arg and/or BH4 (Fig. 7, *A* and *B*). The NADPH oxidation rate under control conditions in the presence of Arg and BH4 (714 ± 23 nmol·mg^−1^·min^−1^) corresponds to a NADP^+^/citrulline stoichiometry of 1.51 ± 0.06, indicative of strong coupling (7). Na_2_S completely blocked NADPH oxidation with an IC_50_ of (1.9 ± 0.3)·10^−5^ m in good accordance with the value observed for citrulline formation (Fig. 1). This indicates that inhibition targets NADPH oxidation without any sign of uncoupling. Interestingly, when Arg and/or BH4 were omitted, conditions under which no irreversible inhibition of citrulline formation occurs (see above), NADPH oxidation was still blocked but at considerably higher concentrations of Na_2_S (IC_50_ values: +Arg/−BH4, (3.3 ± 0.4)·10^−4^ m; −Arg/+BH4, (6.1 ± 1.1)·10^−4^ m; −Arg/−BH4, (9.5 ± 1.4)·10^−5^ m). These observations suggest that H_2_S alone inhibits nNOS reversibly with an IC_50_ of ∼0.1–0.6 mm but that inhibition becomes more pronounced and irreversible in the presence of NO.

To confirm this, we repeated the experiments in which Arg and/or BH4 was omitted in the presence of the NO donor PROLI/NO (Fig. 7*C*). Under these conditions, 30 μm PROLI/NO lowered the IC_50_ to values similar to those observed in the combined presence of Arg and BH4 (IC_50_ values: +Arg/−BH4, (1.5 ± 0.5)·10^−5^ m; −Arg/+BH4, (2.0 ± 0.9)·10^−5^ m; −Arg/−BH4, (1.34 ± 0.19)·10^−5^ m). These results confirm that inhibition by H_2_S is potentiated by NO.

##### Effect of the NO Concentration on Inhibition of nNOS and eNOS by Na_2_S

**FIGURE 8.**
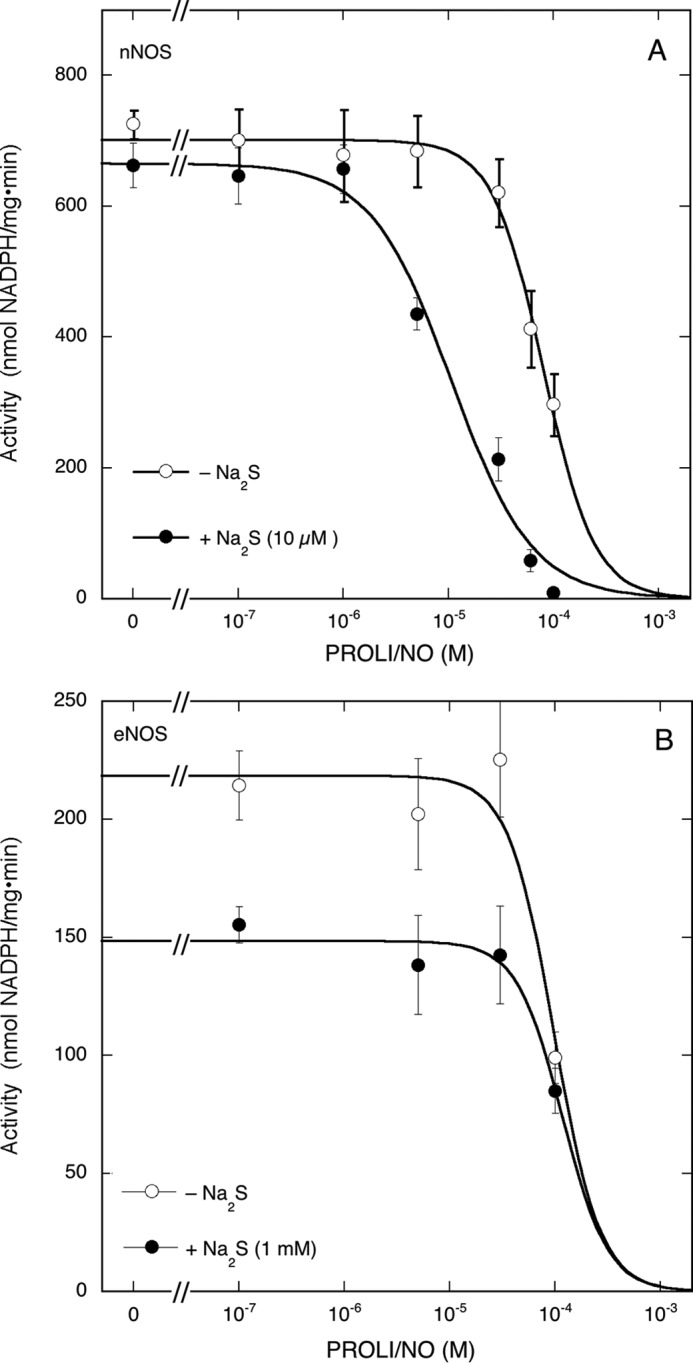
**Effect of PROLI/NO on NADPH oxidation by nNOS and eNOS in the absence or presence of Na_2_S.** *A* shows the effect of PROLI/NO on nNOS-catalyzed NADPH oxidation in the presence of Arg and the absence of BH4 with and without Na_2_S. *B* shows the effect of PROLI/NO on eNOS-catalyzed NADPH oxidation in the presence of Arg and BH4 with and without Na_2_S. Samples (0.25 ml) containing 2.5 μg of nNOS (*A*) or 4 μg of eNOS (*B*), 0.1 mm Arg, 0.2 mm NADPH, 10 μm BH4 (*B* only), 0.5 mm CaCl_2_, 20 μg/ml CaM, 0.2 mm CHAPS, 0.1 mm EDTA, Na_2_S (10 μm in *A*; 1 mm in *B*), and PROLI/NO as indicated in 50 mm TEA (pH 7.4) were incubated for 5 min at 37 °C. Reactions were initiated by the addition of CaM. Data points (*n* ≥ 3) are presented as mean values ±S.E. (*error bars*).

To study the effect of the NO concentration, we measured the rate of NADPH formation at varying PROLI/NO concentrations in the presence of Arg but in the absence of BH4 under which conditions the enzyme does not produce NO. Determination of the effect of the NO concentration is complicated by the fact that Na_2_S alone already inhibits the enzyme (see Fig. 7*B*). Moreover, NO alone will also inhibit NOS activity by binding to the heme (30–32). Therefore we determined the effect of the NO concentration in the absence and presence of 10 μm Na_2_S, a concentration that does not by itself inhibit NADPH oxidation but that becomes inhibitory in the presence of PROLI/NO (Fig. 7, *B* and *C*, *blue* traces). As illustrated by Fig. 8*A*, PROLI/NO inhibited NADPH activity with an IC_50_ of ∼(8.0 ± 0.5)·10^−5^ m in the absence of Na_2_S, which is most likely caused by binding of NO to the heme. In the presence of Na_2_S, the IC_50_ shifted leftward to (1.1 ± 0.2)·10^−5^ m, probably reflecting the (irreversible) effect of NO on H_2_S-induced inhibition.

For comparison, we also looked into the effect of the PROLI/NO concentration in the presence of Na_2_S on eNOS activity. Unlike nNOS, eNOS exhibits greatly reduced NADPH oxidation when either BH4 or Arg is omitted (33). Therefore we decided to include Arg and BH4 in the reaction mixture, and as a consequence, the enzyme already produces NO in the absence of PROLI/NO. We also applied a much higher Na_2_S concentration (1 mm) because at that concentration we earlier observed moderate inhibition of eNOS activity (see Fig. 1). Fig. 8*B* shows that in this case too NADPH oxidation was inhibited by high concentrations of PROLI/NO (IC_50_ = (1.0 ± 0.2)·10^−4^ m). Fig. 8*B* also confirms the moderate effect of 1 mm Na_2_S on NADPH oxidation. However, this weak inhibitory effect was not potentiated by PROLI/NO (IC_50_ = (1.17 ± 0.16)·10^−4^). Taken together, these results demonstrate that NO concentration-dependently potentiates the inhibition by Na_2_S of nNOS but not of eNOS.

In the experiments described above, the effect of NO on H_2_S-induced inhibition of nNOS was clearly concentration-dependent. By contrast, in the experiments of Table 1, similar inhibition was observed at all nNOS concentrations and therefore at all NO concentrations. This suggests that in the studied enzyme concentration range (which corresponded to NO concentrations after 10 min between 2 and 50 μm in the absence of Na_2_S and approximately 10-fold lower concentrations in the presence of Na_2_S) the potentiation by NO of H_2_S-induced inhibition is not affected by its concentration. To corroborate this finding, we determined the effect of the nNOS concentration on the IC_50_ value of Na_2_S. Variation of the nNOS concentration did not affect the IC_50_ values (22 ± 5, 25 ± 6, and 37 ± 3 μm at 1, 5, and 15 μg/ml, respectively), confirming that in this concentration range, which corresponds to uninhibited NO formation rates between 0.37 and 4.3 μm/min, inhibition does not depend on the NO concentration.

##### Effect of Slow H_2_S and NO Donors on nNOS Activity

To study the effect of the rate of H_2_S generation on the inhibition of nNOS, we replaced Na_2_S, which releases H_2_S almost instantaneously, by the slow H_2_S-releasing agent GYY4137 (*t*_½_ ∼ 415 min; Ref. 34). Under full-turnover conditions, *i.e.* in the presence of BH4 and Arg, citrulline formation over a time interval of 25 min was inhibited by GYY4137 with an apparent IC_50_ value of (2.2 ± 0.3)·10^−3^ m (not shown). At that concentration, GYY4137 will release ∼9·10^−5^ m H_2_S in fair agreement with the IC_50_ value obtained with Na_2_S under similar conditions (see Fig. 1).

To study the effect of the NO release rate, we replaced PROLI/NO (*t*_1/2_ ∼ 1–2 s) by SPER/NO (*t*_1/2_ ∼ 1800 s) (35). In the absence of BH4, *i.e.* when the enzyme does not produce NO, SPER/NO potentiated the inhibitory effect of Na_2_S on NADPH formation after 10 min with an apparent IC_50_ value of (1.06 ± 0.17)·10^−4^ m (not shown). At this concentration, SPER/NO will release approximately 24 μm NO in 10 min in good agreement with the values obtained with PROLI/NO (see Fig. 7*C*).

##### Inhibition of nNOS by HNO

**FIGURE 9.**
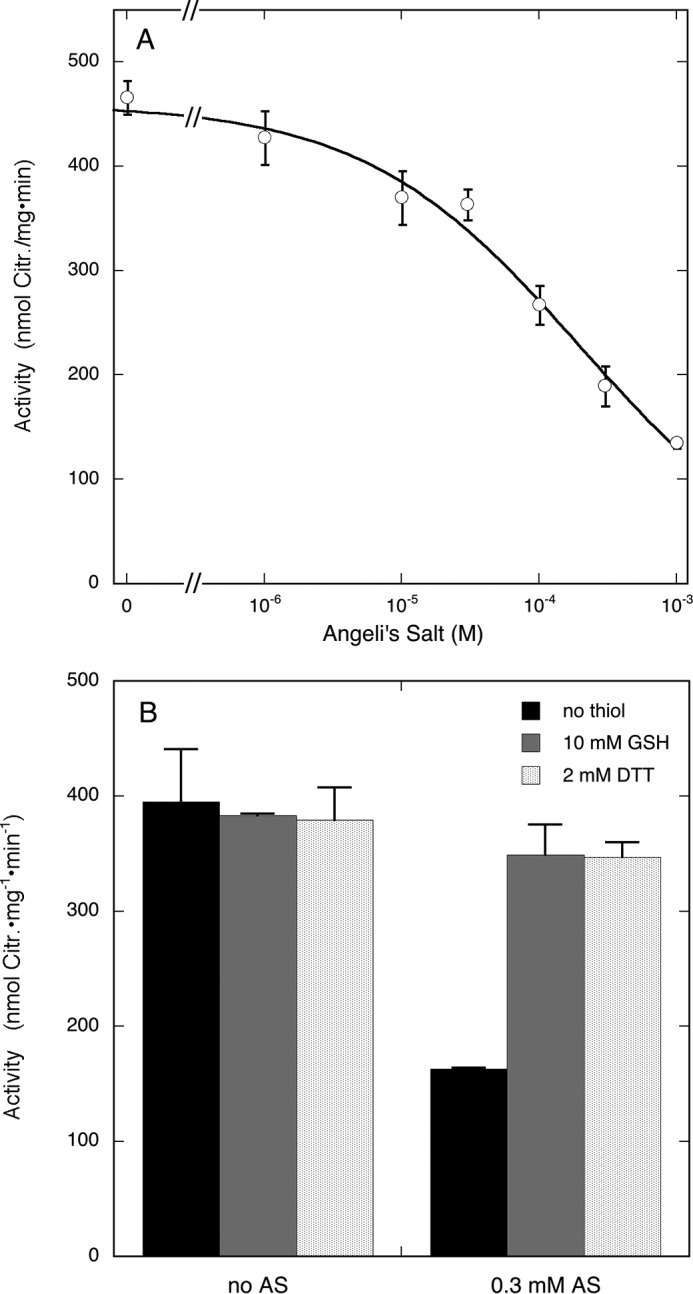
**Effect of Angeli's salt on citrulline formation by nNOS.** *A* shows the concentration dependence of the inhibition by Angeli's salt of nNOS-catalyzed citrulline (*Citr.*) formation. Samples (0.1 ml) containing 0.5 μg of NOS, 0.1 mm [^3^H]Arg (∼60,000 cpm), 0.2 mm NADPH, 5 μm FAD, 5 μm FMN, 10 μm BH4, 0.5 mm CaCl_2_, 10 μg/ml CaM, 0.2 mm CHAPS, 0.1 mm EDTA, and Angeli's salt as indicated in 50 mm TEA (pH 7.4) were incubated for 10 min at 37 °C. Reactions were initiated by the addition of CaM. Data points (*n* = 2) are presented as mean values ±S.E. (*error bars*). *B* shows the effect of thiols on the inhibition by Angeli's salt (*AS*). Experimental conditions were the same as for *A* except for the presence or absence of 10 mm GSH or 2 mm DTT as indicated (*n* = 2).

According to a recent report, the combination of NO and H_2_S regulates vascular tone by the intermediate formation of HNO (36), and it has been reported in the past that HNO is a stronger inhibitor of nNOS than NO (37). To investigate the potential involvement of HNO in the inhibition observed here, we determined the effect of the HNO donor Angeli's salt on citrulline formation by nNOS. As illustrated by Fig. 9*A*, Angeli's salt inhibited nNOS, but the IC_50_ value of (1.9 ± 0.4)·10^−4^ m was considerably higher than that of Na_2_S (see Fig. 1). More importantly, unlike the effect of Na_2_S, inhibition by HNO was completely reversed in the presence of thiols (Fig. 9*B*).

##### Effect of Na_2_S on Dimeric Structure of nNOS and eNOS in the Absence or Presence of NO Synthesis

**FIGURE 10.**
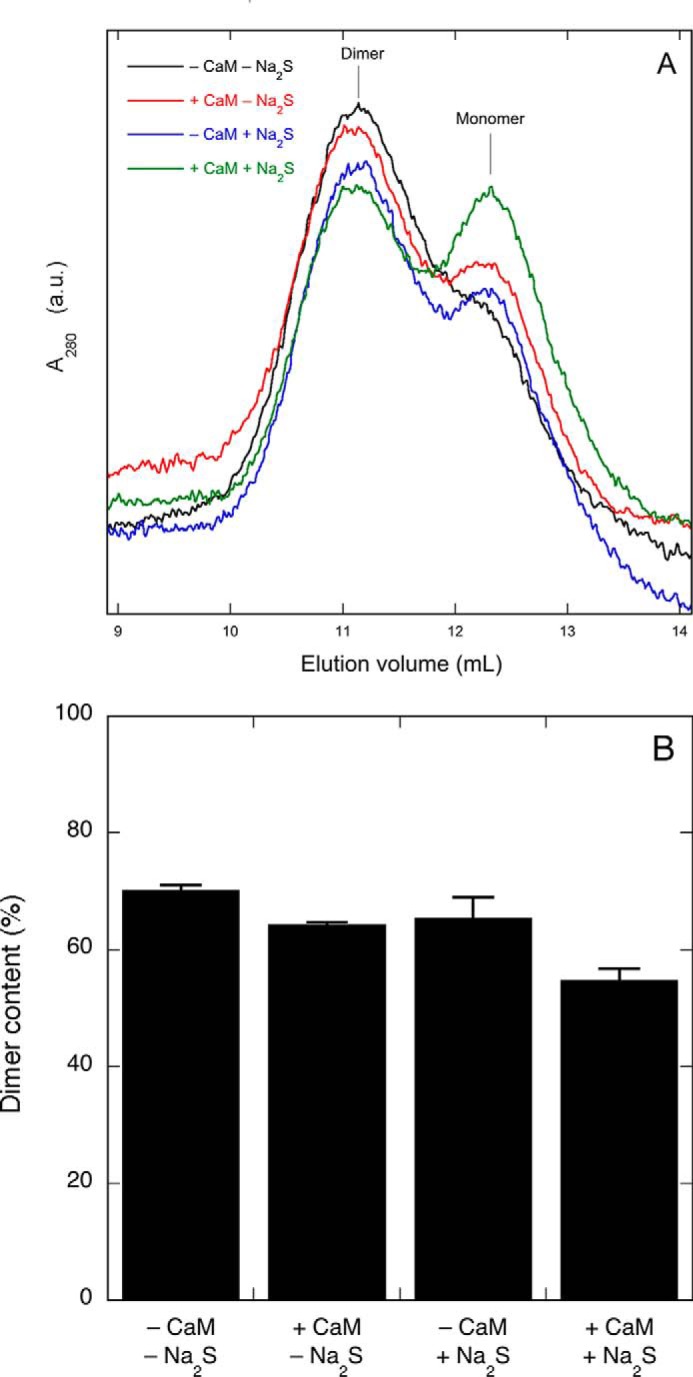
**Effect of NO/H_2_S on the dimer content of nNOS.** *A* shows representative elution profiles of nNOS after preincubation in the presence or absence of CaM and Na_2_S (*black* curve, −CaM −Na_2_S; *red* curve, +CaM −Na_2_S; *blue* curve, −CaM −Na_2_S; *green* curve, +CaM +Na_2_S). *B* shows the corresponding quantification (*n* = 2–3). Preincubation conditions were as follows: 250 μg/ml (1.6 μm) nNOS, 0.1 mm Arg, 0.2 mm NADPH, 5 μm FAD, 5 μm FMN, 10 μm BH4, 0.5 mm CaCl_2_, 0.2 mm CHAPS, 0.1 mm EDTA, 300 μg/ml CaM, and 0.1 mm Na_2_S as indicated in 0.4 ml of 50 mm TEA (pH 7.4) at 37 °C for 10 min. Data are presented as mean values ±S.E. (*error bars*). *a.u.*, absorbance units.

**FIGURE 11.**
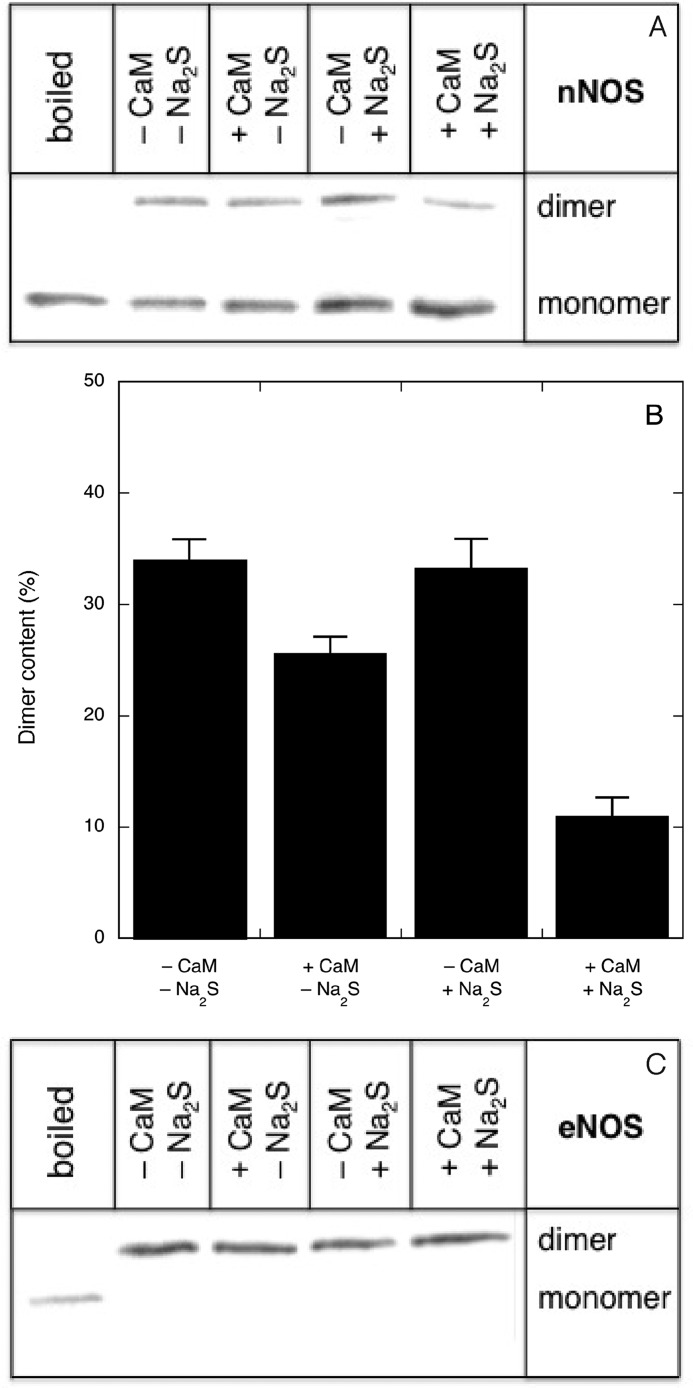
**Effect of Na_2_S on the strength of the dimeric structure of nNOS and eNOS in the absence or presence of NO synthesis.** *A* shows a representative Western blot illustrating the SDS-resistant nNOS dimer/monomer ratio. *B* shows the corresponding densitometric quantification. Data are presented as mean values ±S.E. (*error bars*). *C* shows a representative blot of the effect of Na_2_S on the strength of the dimeric structure of eNOS in the absence or presence of NO synthesis. nNOS (5 μg/ml; 31.3 nm) or eNOS (5 μg/ml; 37 nm) was incubated for 10 min in 0.1 ml of 50 mm TEA (pH 7.4) containing 0.1 mm Arg, 0.2 mm NADPH, 5 μm FAD, 5 μm FMN, 10 μm BH4, 0.5 mm CaCl_2_, 0.2 mm CHAPS, and 0.1 mm EDTA in the absence or presence of 10 μg/ml CaM and/or 0.3 mm Na_2_S at 37 °C. Reactions were terminated by the addition 0.1 ml of chilled 0.125 m Tris (pH 6.8) containing 4% (w/v) SDS, 10% (v/v) 2-ME, 20% (w/v) glycerol, and 0.02% (w/v) bromphenol blue. See “Experimental Procedures” for further details (*n* = 2–4).

To investigate the effect of H_2_S on the dimer content of nNOS, we performed gel filtration chromatography after preincubation under various conditions. Because dimer stability is affected by Arg and BH4 but not by CaM (26), both Arg and BH4 were included in all preincubations, and the effect of NO formation was instead determined by omitting or including CaM. As shown in Fig. 10, after preincubation in the absence of CaM and Na_2_S, the enzyme was mostly (∼70%) dimeric. Preincubation in the presence of CaM or Na_2_S appeared to cause a slight decrease in dimer content, whereas a somewhat larger decrease was observed when CaM and Na_2_S were both present. However, ∼55% of the enzyme was still dimeric even after preincubation under full-turnover conditions. Similar observations were made with low temperature PAGE followed by Western blotting analysis. As shown in Fig. 11, *A* and *B*, Na_2_S alone did not affect dimer stability (33.2 ± 2.6 *versus* 34.0 ± 1.8%), whereas the combination of CaM and Na_2_S reduced the amount of SDS-resistant dimers by more than half (11.0 ± 1.6 *versus* 25.6 ± 1.5%). The dimer/monomer ratio of eNOS was not affected by Na_2_S at all (Fig. 11*C*). Although these results suggest some correlation between dimer strength and NO/H_2_S-induced inhibition, nNOS remained mainly dimeric under conditions that resulted in complete loss of activity, indicating that the main mechanism for inhibition does not involve monomerization.

## Discussion

Inhibition of NOS by H_2_S has been reported previously by Kubo *et al.* (15, 16). In those studies, all three isoforms were inhibited with comparably low potencies (IC_50_ values between 0.13 and 0.21 mm). Furthermore (contrary to what was stated in Ref. 16), inhibition was partly or completely countered by increasing the NADPH concentration and except for iNOS by increasing the BH4 concentration. The reason for these and other discrepancies, the study also reported an IC_50_ for inhibition of nNOS by DTT of 13.2 mm where we previously found 0.16 mm (27), cannot be resolved here but may be related to the use of commercial enzyme preparations with low activity and different experimental conditions (for instance, extremely low concentrations of Arg and CaM and very long incubation times).

The respective mechanisms of inhibition by H_2_S and by NO/H_2_S are unclear. Inhibition is not competitive with any of the substrates or cofactors. Furthermore, inhibition by NO/H_2_S but not by H_2_S alone is irreversible under the present conditions. A puzzling aspect of the present study is the apparent difference in the inhibitory efficiency of nNOS- and PROLI/NO-generated NO. No [NO] dependence was observable down to concentrations as low as 0.5 μg/ml nNOS, which corresponds to a production of NO of ∼0.3 μm after 10 min in the presence of Na_2_S. In contrast, PROLI/NO exhibited an apparent IC_50_ of ∼10^−5^ m. Tentatively, one may ascribe this remarkable difference to the close proximity of the sites of NO formation and H_2_S inhibition in the case of endogenously produced NO.

Cysteinyl side chains are the most likely targets for inhibition by H_2_S. In the presence of an electron acceptor, H_2_S may cause protein *S*-sulfhydration (17, 28). Indeed, *S*-sulfhydration of NOS has been reported recently (38). However, in that study, eNOS was stimulated rather then inhibited by sulfhydration. There have also been several reports on eNOS glutathionylation, which blocked NO synthesis but not NADPH oxidation, resulting in uncoupled catalysis (39–41). By contrast, we are not aware of any study on the glutathionylation or sulfhydration of the neuronal and inducible isoforms. All three NOS isoforms are also inhibited by *S*-nitrosation (42–45), which targets the cysteinyl side chains coordinating the zinc cation that stabilizes the NOS dimeric structure (46, 47). In addition to modification of cysteinyl side chains, it is conceivable that H_2_S directly interferes with NOS zinc binding as has been proposed as a potential inhibitory mechanism in the case of angiotensin-converting enzyme and phosphodiesterase (48, 49). If the interdomain zinc cation or its cysteinyl ligands are indeed the target for inhibition by H_2_S, this would offer a tentative explanation for the remarkable resistance of eNOS to inhibition. Of the three isoforms, eNOS has by far the greatest dimer stability (50). Although the present results indicate that inhibition is not caused by NOS monomerization, it is conceivable that the same forces that stabilize the eNOS dimer also protect the zinc site against inhibition by H_2_S.

Whereas sulfhydration of specific cysteinyl residues might be causing the low affinity reversible inhibition, irreversible inhibition in the presence of NO may involve a product of the reaction between H_2_S and NO. We recently demonstrated efficient nitrosation of GSH and other thiols by NO at submicromolar concentrations (35, 51). A similar reaction with H_2_S would yield HSNO, which in principal might inhibit NOS by transnitrosation of one of the cysteinyl zinc ligands. However, the observation that inhibition was not reversed by thiols or thioredoxin/thioredoxin reductase argues against that possibility. For the same reason, the involvement of HNO can be ruled out as well. In the presence of excess H_2_S, the highly unstable HSNO is rapidly transformed to nitrosopersulfide (SSNO^−^) (17, 18, 52). Conceivably, it is this compound that is responsible for irreversible inhibition of nNOS and iNOS. Although as far as we are aware the p*K_a_* of nitrosopersulfide has not been reported, it is tempting to ascribe the value of 7.3 that we observed for nNOS inhibition to the HSSNO/SSNO^−^ equilibrium. Alternatively, NO or an NO-derived compound may react with the sulfhydrated protein formed by H_2_S in the absence of NO, which would possibly explain the absence of an effect of the NO concentration on inhibition (although not the higher potency of NO/H_2_S compared with H_2_S alone). Clearly, elucidation of the inhibitory mechanism must await identification and characterization of the inhibitory site. To this end, we are currently performing mass spectrometric analysis of the modification of nNOS by NO/H_2_S. Preliminary results suggest that a specific cysteine residue in the reductase domain (Cys^1231^) becomes sulfinated in the presence of H_2_S under turnover conditions.^3^ However, additional studies are required to confirm or refute these observations.

The present results demonstrate that H_2_S completely blocks nNOS activity (coupled and uncoupled) at moderately high concentrations. Importantly, inhibition gets stronger and becomes irreversible under conditions of coupled turnover or when NO is co-administered. Similar effects were observed for iNOS but not for eNOS, demonstrating that inhibition by NO/H_2_S is isoform-specific. There is controversy in the literature on the physiological levels of H_2_S with earlier reports suggesting unrealistically high values (for a review, see Ref. 53), whereas more recent estimates seem to converge on values in the submicromolar or even low nanomolar range (2, 10, 54, 55). Whereas the higher estimates would render the effects observed here physiologically relevant, inhibition by H_2_S alone would be too weak to play a significant role if the lower estimates apply. However, because of its apparent irreversible nature, inhibition by NO/H_2_S might still be relevant. One may speculate that such inhibition could serve a protective role as a negative feedback mechanism in the case of excessive NO/H_2_S production. It will therefore be important to establish whether inhibition by NO/H_2_S remains irreversible in an *in vivo* setting.

In summary, we have observed inhibition of nNOS but not of eNOS that may be physiologically relevant provided that the irreversible character observed here persists under (patho)physiological conditions. If so, these observations may help resolve some of the controversies concerning the impact of H_2_S on NO signaling where both stimulatory and inhibitory effects have been reported.

## Author Contributions

The study was conceived by C. L. H., K. S., B. M., and A. C. F. G. and designed by C. L. H. and A. C. F. G. Data were acquired and analyzed by C. L. H., R. S., K. G., and B. G. and interpreted by C. L. H., A. S., and B. G. The paper was written by C. L. H. and A. C. F. G. and revised by A. S., K. S., and B. M. All authors reviewed the results and approved the final version.

## Footnotes

3 C. L. Heine and B. Gesslbauer, unpublished observations.

## Footnotes

2 The abbreviations used are:

nNOS: neuronal NOS
iNOS: inducible NOS
eNOS: endothelial NOS
CaM: calmodulin
BH4: tetrahydrobiopterin ((6*R*)-2-amino-6-[(1*R*,2*S*)-1,2-dihydroxypropyl]-5,6,7,8-tetrahydropteridin-4(1*H*)-one)
[^3^H]Arg: l-[2,3,4,5-^3^H]arginine hydrochloride
[^3^H]citrulline: l-[2,3,4,5-^3^H]citrulline
PROLI/NO: proline NONOate (1-(hydroxy-*NNO*-azoxy)-l-proline, disodium salt)
SPER/NO: spermine NONOate ((*Z*)-1-[*N*-[3-aminopropyl]-*N*-[4-(3-aminopropylammonio)butyl]amino]diazen-1-ium-1,2-diolate
Angeli's salt: disodium diazen-1-ium-1,2,2-triolate
2-ME: 2-mercaptoethanol
TEA: triethanolamine HCl
Bis-tris propane: 1,3-bis(tris(hydroxymethyl)methylamino)propane.
